# Helical Microstructures of the Mineralized Coralline Red Algae Determine Their Mechanical Properties

**DOI:** 10.1002/advs.202000108

**Published:** 2020-04-21

**Authors:** Nuphar Bianco‐Stein, Iryna Polishchuk, Gabriel Seiden, Julie Villanova, Alexander Rack, Paul Zaslansky, Boaz Pokroy

**Affiliations:** ^1^ Department of Materials Science and Engineering and the Russell Berrie Nanotechnology Institute Technion−Israel Institute of Technology Haifa 32000 Israel; ^2^ Moriah Scientific Consulting Yehiel Paldi St 11 Rehovot 7624811 Israel; ^3^ The European Synchrotron CS 40220 Grenoble Cedex 9 38043 France; ^4^ Department of Restorative and Preventive Dentistry Institute for Dental and Craniofacial Sciences Charité–Universitätsmedizin Berlin Berlin 14197 Germany

**Keywords:** biominerals, algae, calcite, hierarchical structure, mechanical properties

## Abstract

Through controlled biomineralization, organisms yield complicated structures with specific functions. Here, *Jania* sp., an articulated coralline red alga that secretes high‐Mg calcite as part of its skeleton, is in focus. It is shown that *Jania* sp. exhibits a remarkable structure, which is highly porous (with porosity as high as 64 vol%) and reveals several hierarchical orders from the nano to the macroscale. It is shown that the structure is helical, and proven that its helical configuration provides the alga with superior compliance that allows it to adapt to stresses in its natural environment. Thus, the combination of high porosity and a helical configuration result in a sophisticated, light‐weight, compliant structure. It is anticipated that the findings on the advantages of such a structure are likely to be of value in the design or improvement of lightweight structures with superior mechanical properties.

## Graphical abstract

*Jania* sp., an articulated coralline red alga that secretes high‐Mg calcite as part of its skeleton, exhibits a remarkable structure, which is highly porous and reveals several hierarchical orders from the nano to the macroscale. It is shown that its structure is helical and confers the alga with superior compliance that allows it to adapt to stresses in its natural environment.

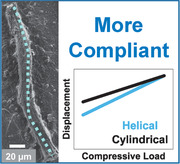

## 1 Introduction

Biomineralization is the science of formation of minerals by living organisms.^[^ ^1^ ^]^ Calcite, the most thermodynamically stable polymorph of calcium carbonate, is an abundant structural component in the skeletal parts of many marine organisms.^[^ ^1^, ^2^, ^3^, ^4^ ^]^ In contrast to synthetic calcite crystals which have clear and flat crystal facets, biogenic calcite crystals possess curved morphologies and shapes.^[^ ^5^, ^6^, ^7^, ^8^ ^]^ These complicated morphologies were previously shown to be obtained by crystallization via a precursor amorphous phase, amorphous calcium carbonate,^[^ ^9^, ^10^, ^11^ ^]^ as evidenced for example by the crystallization path of the sea urchin spine.^[^ ^9^, ^12^ ^]^ It was also shown that by controlled biomineralization, organisms often yield minerals that possess unique and superior properties compared to their synthetic counterparts.^[^ ^13^, ^14^, ^15^, ^16^, ^17^ ^]^

Biominerals are biocomposites composed of inorganic as well as organic components that often play a crucial role in the biomineralization process.^[^ ^18^, ^19^, ^20^, ^21^, ^22^, ^23^, ^24^, ^25^ ^]^ Most of the calcitic biominerals contain some Mg that is incorporated into the calcite lattice,^[^ ^26^, ^27^, ^28^, ^29^ ^]^ with Mg content reaching values as high as 45 mol% as observed in the tooth of the sea urchin *Paracentrotus lividus*.^[^ ^30^, ^31^ ^]^ The transition through an amorphous precursor facilitates the incorporation of large amounts of Mg into the calcite lattice, exceeding the thermodynamically stable Mg concentration at ambient conditions (≈10 mol% of MgCO_3_, although this value is still debatable and might be lower)^[^ ^27^, ^32^ ^]^ to form high‐Mg calcite.

Incorporation of inorganic inclusions, among them Mg, into the crystal structure of calcite is a well‐known strategy exploited by these organisms to improve their mechanical properties.^[^ ^14^, ^30^, ^33^ ^]^ When Mg is incorporated into the calcite lattice it is found in lattice substitution sites, replacing the Ca ions.^[^ ^34^ ^]^ Since the Mg ion is smaller than that of Ca,^[^ ^35^ ^]^ such substitution results in compressive stresses in the crystal. These stresses impede dislocation movements, leading to an increase in hardness.^[^ ^33^, ^36^, ^37^ ^]^ The hardness of both synthetic and biogenic calcite increases with increasing Mg concentration.^[^ ^30^, ^33^ ^]^

Not only Mg incorporation into the calcite lattice, but also the structure of the resulting biomineral has a great impact on its mechanical properties, as reported for many biominerals such as nacre,^[^ ^38^, ^39^ ^]^ the brittlestar *Ophiocoma wendtii*,^[^ ^13^, ^40^ ^]^ the mantis shrimp^[^ ^41^, ^42^ ^]^ as well as in human teeth and bones.^[^ ^43^, ^44^ ^]^ The unique structural organization of these biominerals intensifies their properties relative to those of synthetic minerals.^[^ ^13^, ^33^, ^45^ ^]^

The coralline red algae (phylum Rhodophyta) are prevalent around the world's oceans, where they reside both in shallow and in deep waters^[^ ^46^, ^47^, ^48^, ^49^ ^]^ and are important contributors to the ecology of reefs.^[^ ^50^ ^]^ They are generally divided into two main groups, the crustose and the articulated coralline red algae.^[^ ^47^, ^51^, ^52^ ^]^ Crustose coralline red algae are crusts that adhere to the substrates on which they grow, typically rocks, corals, or other algae.^[^ ^47^, ^48^, ^53^ ^]^ Articulated coralline red algae are branched and grow upright from their substrate, to which they adhere at specific points. For this reason they need to endure outer stresses applied on them by ocean waves and currents.^[^ ^46^, ^54^ ^]^

Coralline red algae secrete high‐Mg calcite as part of their skeleton.^[^ ^55^, ^56^ ^]^ The calcification occurs on cell walls, and an organic phase composed of sulfated polysaccharides serves as a framework for the crystallization.^[^ ^52^, ^57^, ^58^, ^59^, ^60^ ^]^ Sulfated polysaccharides common in coralline red algae are agars and carrageenans.^[^ ^55^, ^57^ ^]^ Alginates, mostly found in brown algae, have also been isolated from coralline red algae.^[^ ^55^, ^57^ ^]^ In a study of the mechanical properties of these algae the researchers examined the uncalcified joints connecting calcified segments of *Calliarthron cheilosporioides* Manza, a species of the coralline red algae that resides in intertidal zone habitats.^[^ ^46^ ^]^ They showed that these uncalcified joints (termed genicula, and formed when calcified cells in the alga are decalcified and restructured^[^ ^61^ ^]^) confer the flexibility needed for the alga to resist outer stresses caused by waves.

However, there is missing information on the role of the calcified segments regarding the algae's mechanical properties. In the present study we investigated the structure of these algae at various length scales from the nano to the macroscale using different high‐end techniques such as synchrotron radiation macro and nanotomography. We found that the coralline red algae possess a porous helical microstructure in which nanocrystals of Mg‐calcite are located around the spiraling pores. In addition, we describe the relationship of the observed structural characteristics of these algae to their mechanical properties. We show, using finite element analysis modeling, that the helical nature of the red algae increases their compliance and therefore their resilience to the outer stresses they experience under water.

## 2 Results and Discussion

Samples of the articulated coralline red alga *Jania* sp. were collected from shallow waters of the Mediterranean Sea near Tel Aviv, Israel.

### 2.1. Phase Identification and Chemical Analysis

**Figure 1:**
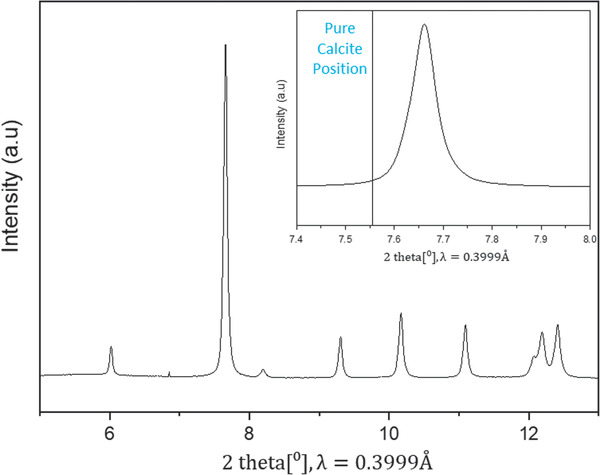
High‐resolution powder X‐ray diffraction (HRPXRD) characterization of *Jania* sp. obtained with synchrotron radiation, λ = 0.3999Å. Inset: {104} peak demonstrating a shift to higher angles than in pure calcite.

The crystalline phase of the alga was identified using high resolution powder X‐ray diffraction (XRD), acquired with synchrotron radiation (radiation wavelength 0.3999Å) at the ID22 of the European Synchrotron Radiation Facility (ESRF, Grenoble, France). Observed positions of the diffraction peaks (**Figure** **1**) were found to be shifted to higher angles than in pure calcite, indicating the shrinkage of the lattice. As an example, the {104} peak shift is shown in Figure 1 (inset). Lattice parameters were calculated using the Rietveld refinement according to the obtained diffraction pattern and were found to be smaller than those of pure calcite (*a* = 4.9305(3) Å; *c* = 16.7904(5) Å). The Mg content in the calcite crystals can be calculated from their lattice parameters, according to previously obtained equations (see Experimental Section). These equations assume that the strains in the crystal are solely due to Mg substitution. We find that the Mg content is 14.1% and 13.2%, by using the equations related to the *a*‐lattice parameter and *c*‐lattice parameter, respectively. The biomineral was identified as Mg‐rich calcite by chemical analysis using inductively coupled plasma optical emission spectroscopy (ICP‐OES), which revealed a Mg content (defined as the ratio Mg/(Ca+Mg)) of 15.38 ± 0.09 at%, confirming that *Jania* sp. is composed of high‐Mg calcite. This was verified by energy dispersive X‐ray spectroscopy (EDS), which revealed a Mg content of 15.3 ± 0.8 at%. From these results it can be determined that the lattice shrinkage (Figure 1) arises from Mg substitution. The difference in the Mg content found from chemical analysis and from XRD analysis can be explained by the presence of additional strains in the sample, that are not taken into account in the XRD analysis.

### 2.2. Structure and Nanostructure

**Figure 2:**
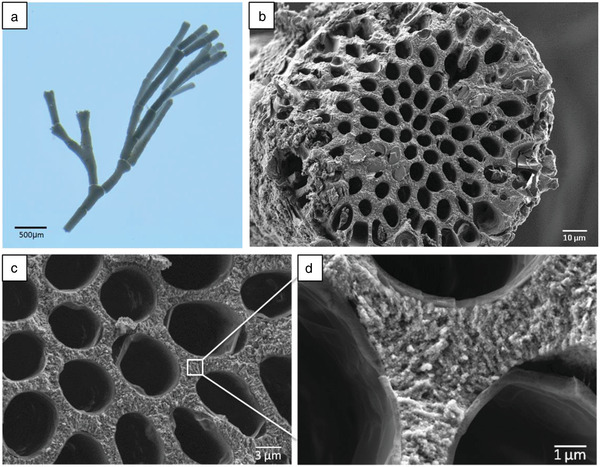
a) Macrostructure of *Jania* sp. examined by optical microscopy. b) Cross section of *Jania* sp. examined by HRSEM. c) Higher magnification of the cross section showing mineralization on cell walls. d) Nanometric crystals comprising the cell walls.

The morphological organization of *Jania* sp. was studied by optical microscopy (**Figure** **2**a), which shows its branched appearance. High‐resolution scanning electron microscopy (HRSEM) of the alga's cross section, shown in Figure 2b, reveals a highly porous structure with mineralization on cell walls. Given this high porosity, specific properties such as strength per weight are expected to be enhanced. Higher magnification of this cross section (Figure 2c) confirms that mineralization occurs on the cell walls. The high‐Mg calcite crystals composing the structure (Figure 2d) are nanometric and are radially aligned.

Secondary electron imaging coupled with back‐scattered electron (BSE) imaging of the alga's cross section at higher magnification (Figure S1a,b, Supporting Information) reveals that crystallization probably occurs on a second phase (template), which shows a darker contrast in the BSE image than that of the mineralized phase indicating that it is lighter, and most probably organic. Coupled thermogravimetric analysis−mass spectroscopy (TGA‐MS) (Figure S1c, Supporting Information) verified this assumption, and according to the MS, at a temperature of ≈290 °C organics are indeed detected. From the corresponding loss of weight of the sample, the organic content was estimated to be about 10.7 wt%.

**Figure 3:**
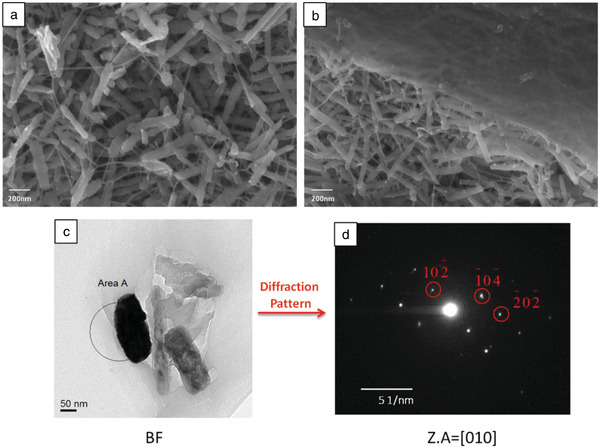
a) Nanoscale investigation of *Jania* sp. surface morphology showing that organic fibers connect the surface crystals and b) an organic layer covers the surface crystals. c) TEM bright‐field (BF) image of a surface crystal. d) Diffraction pattern obtained from “Area A” marked in (c).

The surface morphology of *Jania* sp. is depicted in **Figure** **3**. The surface crystals are rods of a few hundred nanometers long and with nanometric diameters of the order of ≈50 nm. Fibers connect the rod‐shaped crystals (Figure 3a), probably enhancing the elasticity and flexibility of the structure's surface so that it can endure the outer stresses applied on it. In addition, an organic layer covers the alga's surface to protect it from the environment (Figure 3b). To conduct a transmission electron microscopy (TEM) investigation of one of the surface crystals, we obtained a bright‐field image of a crystal (Figure 3c) and selected a diffraction area from the area marked in the image as “Area A”. Since this was a spot diffraction (Figure 3d) we could reasonably assume that it was a single crystal. Diffractions from various zone axes (Z.A) were simulated using the calculated lattice parameters obtained by Rietveld refinement. The simulation shows that the Z.A is [010].

The surface morphology is presumably related to the alga's mechanical properties. The nanometric size of the crystal diameter will prevent the initiation of intra‐crystal cracks, because crystals at the nanometric‐length scale been shown to behave like perfect crystals.^[^ ^62^ ^]^ Furthermore, the meshwork organization of these crystals is believed to improve the structure's mechanical properties, resembling findings previously described for some animal nests.^[^ ^63^, ^64^ ^]^

**Figure 4:**
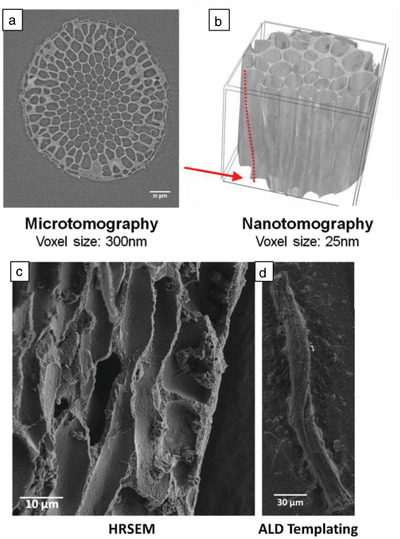
a) Cross section of *Jania* sp. imaged at ID19 of the ESRF using X‐ray microtomography. b) 3D reconstruction of *Jania* sp. based on high‐resolution X‐ray nanotomography performed at ID16B of the ESRF showing the helical microstructure discovered in *Jania* sp. The red dashed line and red arrow highlight a spiraling pore edge. Sample height is 54 µm. c) Longitudinal cross section of *Jania* sp. imaged by HRSEM. A helix is observed in the cross section. d) Alumina replica of one of the *Jania sp*. inner pores obtained via ALD process, imaged by HRSEM.

The 3D microscopic structure of *Jania* sp. was studied by X‐ray microtomography, with a voxel size of 300 nm, at the ID19 beamline of the ESRF. The structure of *Jania* sp. mineralized phase was again observed to be porous (**Figure** **4**a). Using image processing (ImageJ), pore size was found to be of the order of a few microns and the porosity was evaluated as being as high as 64 ± 1 vol%. Another prominent characteristic was that closer to the alga's surface, the alga's mineralization on cell walls is thicker than near its inner part. Since stresses applied on the alga have a greater impact on its outer than on its inner surface, we believe that this feature exists to enhance the mechanical properties of *Jania* sp.

X‐ray nanotomography of *Jania* sp. was also performed at the ESRF's ID16B beamline, where a higher resolution with voxel size of up to 25 nm can be obtained. The obtained 3D reconstruction of the mineralized phase in *Jania* sp. is presented in Figure 4b. The results clearly demonstrate that the microstructure of high‐Mg calcite is helical, rather than cylindrical, an observation which to our knowledge has not been previously reported in these types of algae. Additionally, this surprising helical structure is notable when tracking a single pore along the alga's cross section and observing its rotation (see Movie S1, Supporting Information) This structure is believed to be oriented by the organic phase (see Figure S1, Supporting Information). We note that helical configurations were previously identified in nature mostly as fibers,^[^ ^65^ ^]^ rather than mineralized microstructures as revealed in this study.

**Figure 5:**
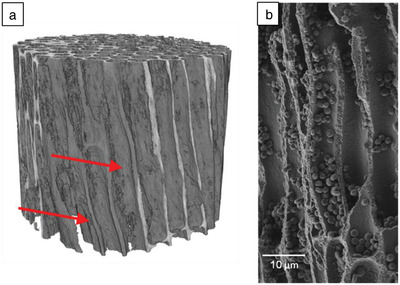
a) 3D reconstruction of *Corallina* sp. based on high‐resolution X‐ray nanotomography performed at ID16B of the ESRF revealing its helical microstructure; voxel size 35 nm. The red arrows highlight spiraling pore edges. b) Longitudinal cross section of *Corallina* sp. imaged by HRSEM.

The helical microstructure of *Jania* sp. was also observed when longitudinal cross sections of the alga were examined by HRSEM (Figure 4c). Yet another proof of this remarkable helical microstructure was obtained when subjecting the alga to alumina deposition via atomic layer deposition (ALD). Due to the alga's high porosity and the usage of gaseous phase precursors, the alumina covered the inner part of the structure and filled the pores. Later, the mineralized phase of the alga was dissolved and the resulting alumina structures were examined by HRSEM. Figure 4d shows one of the obtained structures, clearly demonstrating the helical organization of the alga's inner pores. Based on the HRSEM results and the ALD experiments, the average pitch of the helices is 67 ± 20 µm. Investigation of *Corallina* sp., another species of the coralline red algae, disclosed the same helical microstructure as in *Jania* sp. (**Figure** **5**), indicating that the presence of helical microstructures in coralline red algae is a widespread phenomenon. The observation of the helical microstructure in different coralline red algae strengthens the notion that it is not an arbitrary element yet a structural element with a specific function. We believe that the helical nature of the pores constitutes an additional hierarchical element to the alga's structure to control its mechanical properties.

### 2.3. Modeling of the Mechanical Properties

**Figure 6:**
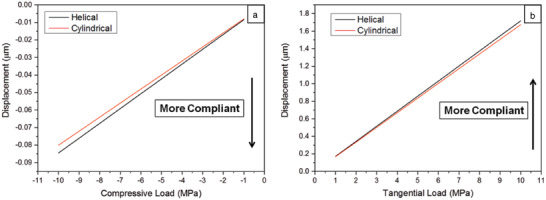
Results of finite element analysis. a) Displacement versus load curves for helical and cylindrical voids under compressive load. b) Displacement versus load curves for helical and cylindrical voids under tangential load.

We used finite element analysis to study the effect of the alga's structure on its mechanical properties. Two types of models were examined, one with cylindrical voids and the other with helical voids (see Figure S2A, Supporting Information). To retain the same volume fraction, the number of voids in the two models differs. The models were examined under two types of loads, compressive (buckling) and tangential (Figure S2B, Supporting Information). Both models were kept fixed to the bottom surface. **Figure** **6** shows higher compliance of the helical microstructure than the cylindrical microstructure in both cases: the helical microstructure was 17% more compliant under the compressive load and 3% more compliant under the tangential load. Since the alga is branched, the outer stresses applied on it by the waves in the sea are a mixture of compressive and tangential loads, and the above results represent the upper and lower limits of the differences in compliance between the two models. The higher compliance of the helical microstructure, in addition to its flexible joints, is most probably what enables the alga to adapt to the waves and avoid failure.

## 3 Conclusion

The results of this study demonstrate the intricate structure and nanostructure of *Jania* sp., a species of the coralline red algae. We found that this alga has a sophisticated low‐weight, high‐strength structure, enabling it to endure outer stresses applied by its natural habitat. Both the structure and the nanostructure of *Jania* sp. are believed to have acquired intensified mechanical properties by virtue of their various compositional and structural characteristics from the nano to the macroscale. Those properties are further enhanced because of the structure's high degree of porosity. The mechanisms strengthening the alga are the chemical composition of high‐Mg calcite leading to compression stresses within the crystal lattice, thickening of the mineralized cell walls closer to the alga's surface than to its inner part, and the morphology and organization of the surface crystals. An extraordinary feature is its helical microstructure, which increases the alga's compliance by allowing it to adapt to the stresses imposed by the waves of the sea. To our knowledge this is a first report of such a helical structure. We believe that this finding is of great importance in deepening our understanding of nature's designs, and is potentially of major significance for the development of new low‐weight, high‐compliance structures.

## 4 Experimental Section

##### Sample Collection

Samples of the coralline red algae *Jania* sp. and *Corallina* sp. were collected from shallow waters of the Mediterranean Sea near Tel Aviv, Israel. Samples were stored in ethanol prior to analysis.

##### High‐Resolution Powder XRD

Crystalline phase was identified by means of high‐resolution powder XRD (HRPXRD) using synchrotron radiation (wavelength 0.3999Å) at the ID22 beamline of the ESRF, Grenoble France. The sample was placed in a glass capillary tube and spun during measurement to avoid effects of preferred orientation.

##### Data Analysis

Lattice parameters were calculated using Rietveld refinements in GSAS‐II software. Mg content (defined as the Mg/(Ca+Mg) ratio) was calculated according to Zolotoyabko et al.,^[^ ^66^ ^]^ assuming no distortions in the unit cell except for those caused by Mg substitution.

##### Inductively Coupled Plasma Optical Emission Spectroscopy

ICP‐OES was performed using an iCAP 6300 Duo ICP‐OES spectrometer (Thermo Scientific) for elemental analysis. Samples were weighed before analysis, immersed in 0.6 m HCl, and placed on a rocking table for 24 h to dissolve the Mg calcite.^[^ ^27^ ^]^ Later, insoluble organics were separated from the solution and their weight was subtracted for accurate analysis.

##### Thermogravimetric Analysis‐Differential Scanning Calorimetry−Mass Spectroscopy

Coupled TGA‐MS was performed (the TGA was performed using the Labsys evo TGA/STA‐EGA and MS using the Hiden Quantitative Gas Analyzer). TGA provided a measurement of the organic content within the sample. The coupled MS allows detection of the different phases. To measure a sample of *Jania* sp. with organics the samples were heated from room temperature to 110 °C at a rate of 5 °C min^−1^. The samples were kept at 110 °C for 30 min to allow water residuals to evaporate, then heated to 750 °C at a rate of 5 °C min^−1^ and finally cooled back to room temperature. Measurements were obtained in an environment of 20% oxygen in argon (synthetic air) with an empty crucible as a reference.

##### Optical Microscopy

Optical microscopy was performed using an Olympus stereo microscope SZX7.

##### High‐Resolution Scanning Electron Microscopy

The microstructure and surface morphology of the sample were examined by HRSEM using a Zeiss Ultra‐Plus FEG‐SEM. Prior to measurements the samples were carbon coated.

##### Energy‐Dispersive X‐Ray Spectroscopy

EDS was performed using an Oxford Silicon Drift Detector EDS installed in a Zeiss Ultra‐Plus FEG Silicon Drift Detector SEM. A 10‐kV beam was used for the measurements.

##### Transmission Electron Microscopy

TEM was used to verify that the surface crystals of the alga were single crystals.

##### Microtomography and Nanotomography

Measurements were carried out using synchrotron radiation in beamlines ID19 and ID16B of the ESRF, Grenoble France. Samples were rotated during the measurements and projections were collected at various angles. For nanotomography, projections were collected at four distances from the detector. Later these projections were analyzed and reconstructed using different in‐house algorithms to obtain a 3D volume of the sample. Microtomography was performed at the ID19 beamline with energy of 26.3 keV and a camera pixel size of 300 nm. Nanotomography was performed at the ID16B beamline to obtain higher resolution. *Jania* sp. was measured at 29.6 keV with a camera pixel size of 25 nm. *Corallina* sp. was measured at 17.5 keV with a camera pixel size of 35 nm.

##### Atomic Layer Deposition

Alga templating with alumina was performed by means of ALD. The system used for these experiments was a Picosun R200 PE ALD Advanced‐ALD system. Specimens were glued to Si wafers (one alga frond on each wafer) and placed in the ALD chamber, then coated with an alumina layer ≈100 nm thick. Because the morphology of the algae is intricate, the stop‐flow process was used to obtain optimal dense and conformal alumina coverage. In this process the precursor was introduced at the surface and was then left in the chamber for a predetermined time before being purged. Thus, use of this method allowed a longer period of exposure of the precursors to the available surface sites. ALD was performed at 200 °C with 300 cycles of each of the two precursors, trimethylaluminium and H_2_O. Later, 0.6 m HCl was dropped onto the coated algae to dissolve the Mg‐calcite without affecting the alumina templating, and the structures obtained were imaged by HRSEM.

##### Statistical Analysis

The average pitch of the helices, based on HRSEM results and ALD experiments, was calculated on the basis of 10 measured pitches in the samples.

##### Finite Element Analysis

For numerical simulations finite element analysis was used. Two types of models were compared, one with cylindrical voids and the other with helical voids (pitch of 100 µm), under two modes of loads, compressive (buckling) and tangential loads in the range of 1−10 MPa. The number of voids in the two models differed in order to retain the same volume fraction. Both models were kept fixed to the bottom surface.

## Conflict of Interest

The authors declare no conflict of interest.

## Supporting information

Supporting Information

Supplemental Movie 1
