# Supplementary material for: Helical Microstructures of the Mineralized Coralline Red Algae Determine Their Mechanical Properties
Source: Adv Sci (Weinh). 2020 Apr 21;7(11):2000108. doi: 10.1002/advs.202000108 (PMC7284203; doi:10.1002/advs.202000108)
Supplement: Supplementary file 1 — Supporting Information [file ADVS-7-2000108-s001.pdf]

Copyright WILEY-VCH Verlag GmbH & Co. KGaA, 69469 Weinheim, Germany, 2020.

ADVANCED SCIENCE Open Access

Supporting Information

for *Adv. Sci.,* DOI: 10.1002/advs.202000108

Helical Microstructures of the Mineralized Coralline Red Algae Determine Their Mechanical Properties

Nuphar Bianco-Stein, Iryna Polishchuk, Gabriel Seiden, Julie Villanova, Alexander Rack, Paul Zaslansky, and Boaz Pokroy**

Supporting Information

Helical Microstructure of Mineralized Tissue in Coralline Red Algae Determines their Mechanical Properties

*Nuphar Bianco-Stein, Iryna Polishchuk, Gabriel Seiden, Julie Villanova, Alexander Rack, Paul Zaslansky, Boaz Pokroy**

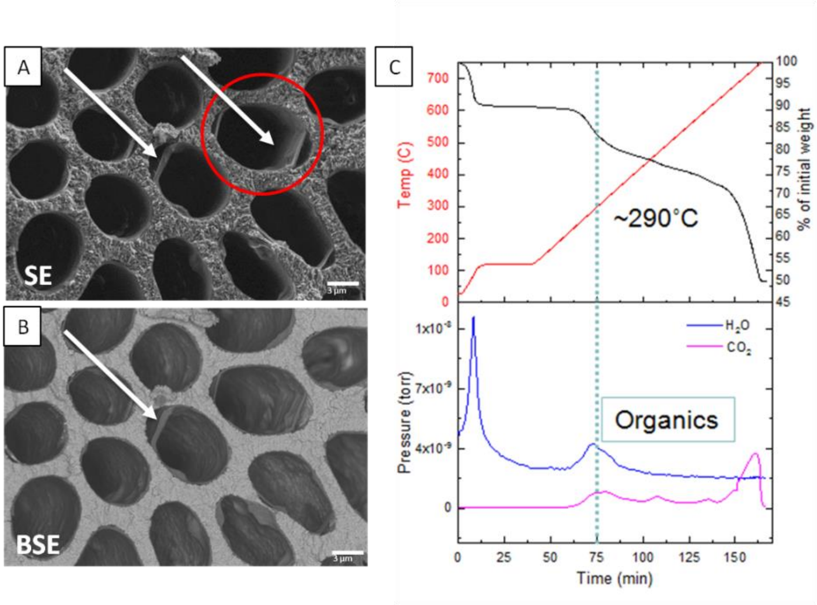

Figure S1. Characterization of the organic phase a) Secondary electron image of a cross section of *Jania* sp. at higher magnification. b) Back-scattered electron image of the area shown in (A), indicating the presence of at least two different phases. c) Coupled TGA-MS.

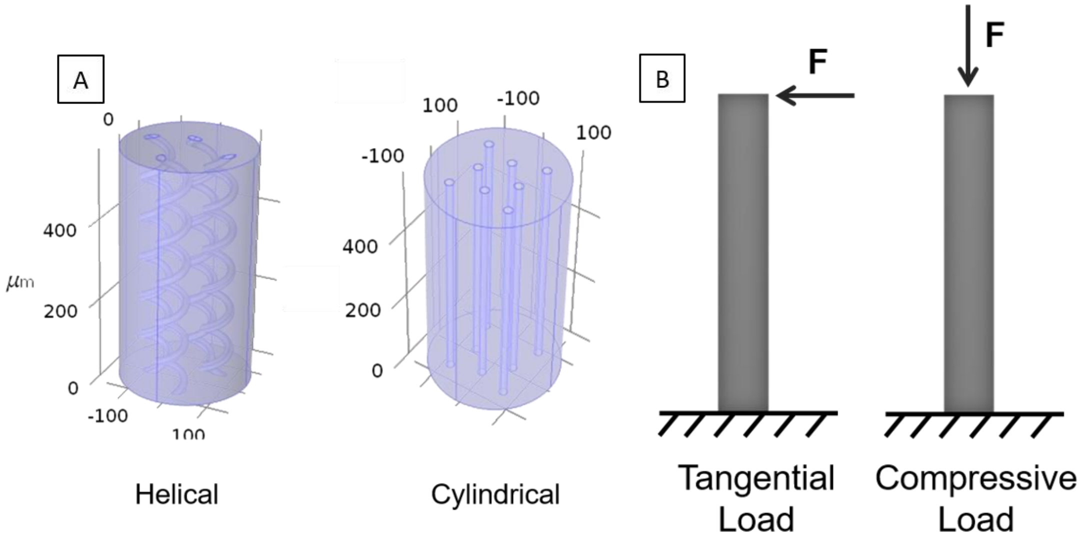

Figure S2. a) Models studied with finite element analysis. b) Modes of load used for the analysis.

Movie S1. Cross section of *Jania* sp. imaged at ID19 of the ESRF using X-ray microtomography along 75µm of the alga displaying its helical pores.
